# Mapping the Oral Microbiome’s Role in Periodontal Disease Progression: A Systematic Review

**DOI:** 10.7759/cureus.110078

**Published:** 2026-06-01

**Authors:** Shaan R Patel, Vishnu S Vundamati, Riyana R Patel, Nisa Eriskin, Caiden J Friedrich, Brielle Tila-Cohen, Daniel Tupikin, Ashritha S Pidikiti, Kabir D Lall, Harvey N Mayrovitz

**Affiliations:** 1 Biology, Nova Southeastern University, Davie, USA; 2 Osteopathic Medicine, Nova Southeastern University Dr. Kiran C. Patel College of Osteopathic Medicine, Davie, USA; 3 Public Health, Nova Southeastern University, Davie, USA; 4 Osteopathic Medicine, Lake Erie College of Osteopathic Medicine, Bradenton, USA; 5 Dentistry, Nova Southeastern University, Davie, USA; 6 Medical Education, Nova Southeastern University Dr. Kiran C. Patel College of Allopathic Medicine, Davie, USA

**Keywords:** aggregatibacter actinomycetemcomitans, functional dysbiosis, microbial interactions, oral microbiome, periodontal disease, porphyromonas gingivalis, tannerella forsythia, treponema denticola

## Abstract

Periodontal disease is an inflammatory condition characterized by progressive destruction of the tooth-supporting tissues and a shift from a symbiotic to a dysbiotic oral microbial community, rather than by a single pathogen. This review aimed to synthesize current evidence on how alterations in microbial composition, community structure, and functional activity contribute to periodontal disease severity and progression.

A comprehensive literature search across four databases (PubMed, Web of Science, Google Scholar, and Embase) was conducted. Studies were included if they were peer-reviewed, human studies published between 2000 and 2026, and met the predefined inclusion and exclusion criteria. Twenty-two articles met these criteria and were analyzed for relationships between microbial patterns and clinical peritoneal outcomes.

Across the studies reviewed, periodontal disease severity was consistently associated with compositional shifts in the oral microbiome rather than changes in overall microbial diversity or bacterial load. Increased prevalence and abundance of red-complex organisms, including *Porphyromonas gingivalis*, *Tannerella forsythia*, and *Treponema denticola*, were strongly associated with worsening clinical parameters, whereas *Aggregatibacter actinomycetemcomitans *showed a stronger association with aggressive disease phenotypes. Functional analyses further revealed enrichment of inflammatory and metabolic pathways, which support the concept of functional dysbiosis as a factor influencing tissue destruction. Interventions that modified local ecological conditions or host-microbe interactions demonstrated improved microbial profiles and clinical outcomes.

These findings reinforce the idea that periodontal disease management is not just about targeting a single pathogen; it should focus on restoring microbial homeostasis and regulating the host's inflammatory response. Adopting this approach will help to create a more effective and personalized treatment strategy for the patient that will likely improve their symptoms, help prevent periodontal disease progression, and reduce their risk of developing complications associated with chronic oral inflammation.

## Introduction and background

Periodontal disease is a widespread chronic inflammatory condition that affects the supporting structures of the teeth and remains a leading cause of oral morbidity worldwide. It arises from a complex interaction between microbial communities and the host immune response, ultimately leading to tissue destruction and, if left untreated, potential tooth loss. While early research focused primarily on the role of plaque accumulation, modern research has shifted toward recognizing the importance of the oral microbiome as a dynamic, interactive ecosystem [1]. This perspective emphasizes that disease is triggered not by a single pathogen, but by a shift in the entire microbial community that disrupts the balance between health and inflammation.

In a healthy oral environment of symbiosis, microbial communities exist in a state of balance with the host. However, periodontal disease is characterized by a transition from symbiosis to dysbiosis, in which pathogenic species increase and microbial interactions are altered. It is important to note that research has shown that overall microbial diversity often remains relatively stable, whereas shifts in microbial composition are strongly associated with disease states [2]. This provides reason to believe that the progression of periodontal disease is more strongly influenced by the specific types of bacteria present rather than the total bacterial load.

Key periodontal pathogens, particularly members of the red complex (*Porphyromonas gingivalis*, *Tannerella forsythia*, and *Treponema denticola*), consistently show increased prevalence and abundance in diseased sites and are closely associated with clinical indicators such as probing depth and attachment loss. Additionally, *Aggregatibacter actinomycetemcomitans *has been implicated more prominently in aggressive forms of the disease, indicating variability in microbial profiles across disease phenotypes [3].

Thorough research has been conducted to explain the severity and progression of periodontal disease through the lens of microbial dysbiosis, focusing on key pathogenic species, shifts in microbial communities, and their relationship with clinical outcomes. Although there is a growing body of research on periodontal disease, gaps remain in our understanding of how changes in the oral cavity's microbial populations affect disease progression. The research fails to clarify whether specific bacteria or the overall microbial diversity of the oral cavity affects periodontal disease. The purpose of this review is to analyze current research that focuses on microbial community structures, the composition of microbes in the mouth, and the functional characteristics of the bacteria, and see how they relate to periodontal disease severity and progression. By addressing the research gap, this review aims to identify microbial patterns and underlying mechanisms associated with clinal outcomes, ultimately improving understanding and targeted treatment of periodontal disease.

## Review

Methods

Search Strategy and Information Sources

A comprehensive search was conducted using the following electronic databases: PubMed, Google Scholar, and Embase. The search was conducted using a predefined list of controlled vocabulary terms and keywords. Search terms included: “subgingival microbiota,” “oral microbial dysbiosis,” “periodontitis,” “red complex bacteria,” “Tannerella forsythia,” “Treponema denticola,” “Aggregatibacter actinomycetemcomitans,” and “Porphyromonas gingivalis.” These descriptors were applied using the standard Boolean operators (AND, OR, NOT) in accordance with database search engine mechanics to capture as many relevant articles as possible. The search methodology was modified in accordance with the specific criteria of every database.

Eligibility Criteria

Initial data screening was performed independently by six authors, and articles that met the inclusion criteria were included in the preliminary review. Table 1 summarizes the inclusion and exclusion criteria utilized during the initial screening process. 1,316 articles were identified in the initial screening. Utilizing the Rayyan software (Rayyan, Cambridge, USA), 258 duplicates were detected. After eliminating duplicate articles, the authors were left with 1058 articles.

**Table 1:** Inclusion and exclusion criteria employed during the search of databases.

Article Inclusion Criteria	Article Exclusion Criteria
English language	Animal and cellular studies
Human and in vitro studies	Studies without data on patient samples or microbial predictors
Gender and age: Any gender 18 years old or older	Studies with a sample size of less than 10 participants per group
Study type: retrospective cohort studies, case control studies, cross-sectional studies, and randomized controlled trials	Study type: systematic reviews, meta-analyses, case reports, grey literature, conference abstracts, editorials, commentaries, pediatric studies, or studies involving pregnant individuals
Any geographic location	Studies prior to the year 2000
Studies discussing changes in oral microbiota and mechanisms towards the onset of periodontal disease	Lack of full-text availability, duplicate publications, or overlapping datasets

Data Extraction and Management

Following duplicate removal, 1,058 records underwent article screening to assess relevance to periodontal disease progression and oral microbiome composition. At this stage, 1,015 articles were excluded due to clearly identifiable methodological or scope-related limitations. The most frequent reason for exclusion was a lack of direct relevance to periodontal disease progression or severity (n=376), as many studies focused on unrelated oral conditions or general microbiome descriptions without clinical periodontal outcomes. A substantial number of records were excluded for lacking clear or appropriate microbiome methodology (n=278), including studies without sequencing-based analyses, microbial outcome measures, or subgingival sampling. Additional exclusions included articles that didn’t provide explicit microbiome characterization (n=189), preventing assessment of microbial composition shifts relevant to disease onset or progression.

**Figure 1:**
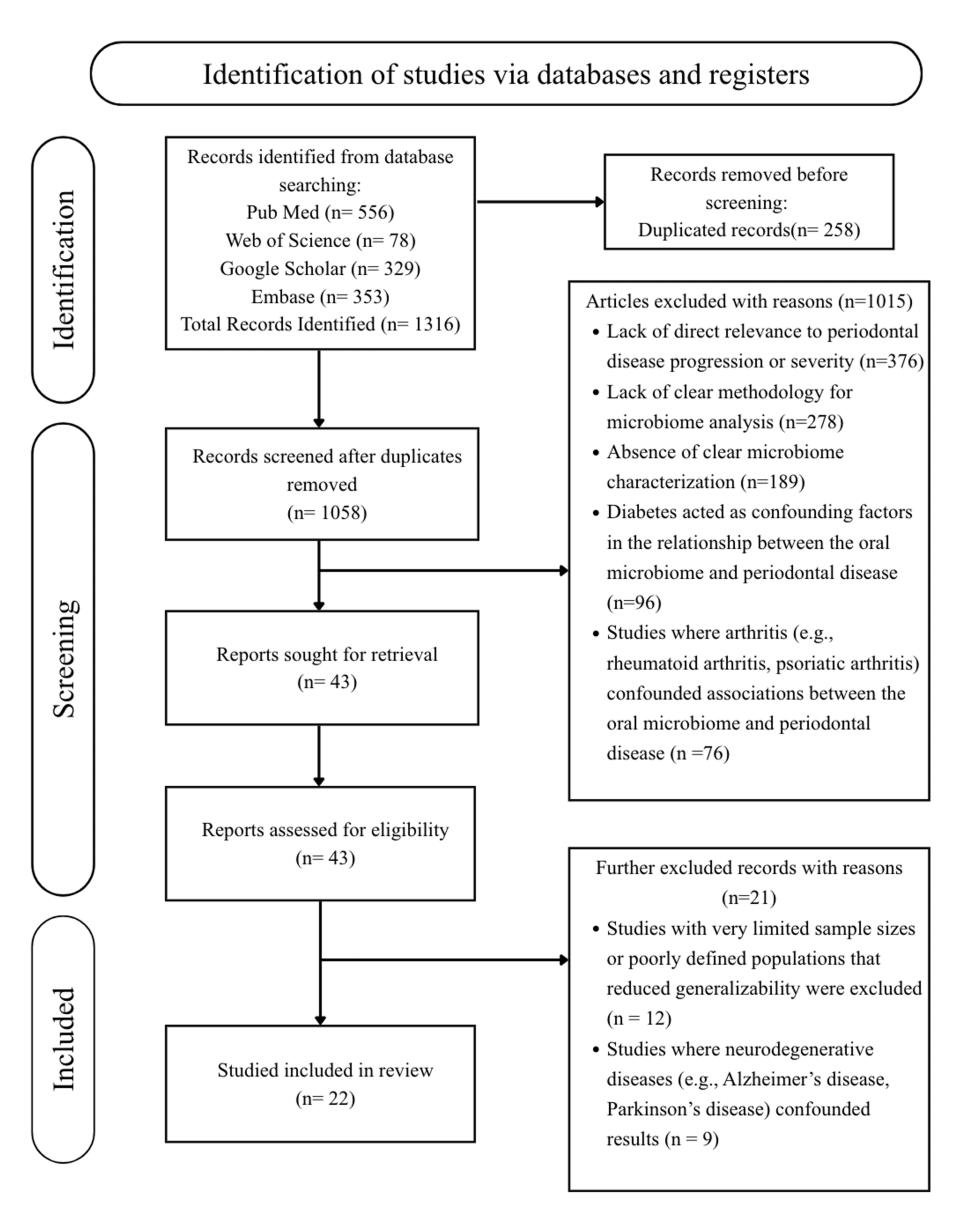
PRISMA diagram Flow diagram created by the authors reflecting the study's selection process in accordance with PRISMA guidelines [4]. PRISMA: Preferred Reporting Items for Systematic Reviews and Meta-Analyses.

Further exclusions during initial screening were driven by confounding clinical variables that obscured the relationship between oral microbiota and periodontal disease. Studies in which diabetes was a primary modifying factor (n=96) were excluded because of its well-established independent impact on both immune regulation and microbial profiles. Similarly, investigations centered on systemic inflammatory conditions such as rheumatoid arthritis or psoriatic arthritis, n=76, were removed as inflammatory confounding limited causal interpretation of microbiome-periodontitis interactions. Collectively, these exclusion criteria ensured that the remaining 43 articles retrieved maintained a focused methodological alignment with the review’s objectives. Subsequent full-text screening resulted in the exclusion of 21 additional studies due to limited sample size or confounding neurodegenerative conditions. Studies with fewer than 10 participants per group were excluded to minimize the impact of underpowered analyses and improve the reliability of microbiome associations. This screening process yielded 22 studies, which are included in the final synthesis, in accordance with the PRISMA (Preferred Reporting Items for Systematic Reviews and Meta-Analyses) guidelines (Figure 1) [4].

Results

Table 2 summarizes the main features of the studies included in this systematic review, providing a structured overview of the current evidence on the oral microbiome in periodontal disease. Each study is organized by key methodological components, including study design, sample size, microbiological methods, and primary findings relevant to disease severity, progression, or microbial dysbiosis. The “Study Design” column categorizes investigations to facilitate comparison among cross‑sectional, experimental, clinical, and randomized controlled studies, while the “Sample Size” column distinguishes participant numbers and, where applicable, the allocation to experimental versus control groups. To further enhance transparency, a dedicated column summarizes sample collection and analytical approaches, highlighting methodological variability across sequencing‑based, culture‑based, and molecular techniques.

**Table 2:** Summary of microbiome studies on periodontitis included in this review N = number of participants; Exp = experimental group; Ctrl = control group; ARG = antibiotic resistance genes; “Red complex” = *P. gingivalis*, *T. forsythia*, *T. denticola;* rRNA = ribosomal RNA; qPCR = quantitative polymerase chain reaction; *P. gingivalis *= *Porphyromonas gingivalis;* *T. denticola *= Treponema denticola; *T. forsythia *= *Tannerella forsythia*; *A. actinomycetemcomitans* = *Aggregatibacter actinomycetemcomitans*; *S. cristatus* = *Streptococcus cristatus*; *P. nigrescens* = *Prevotella nigrescens*; *F. nucleatum* = *Fusobacterium nucleatum*.

Study	Study Design	Sample Size	Sample Collection and Analysis	Findings
Doğan et al., 2003 [5]	Cross-sectional	N (Exp / Ctrl): 30 / 30	Subgingival plaque samples collected. Bacterial load measured by culture.	Periodontitis showed higher levels of key pathogens (*P. gingivalis*, *A. actinomycetemcomitans*), with greater abundance in severe disease and strong interspecies associations.
Jung et al., 2024 [6]	Cross-sectional	N (Exp / Ctrl): 39 / 12	Subgingival plaque and saliva samples were analyzed by 16S rRNA sequencing.	Salivary microbiota closely reflected subgingival microbial shifts, supporting salivary microbiota as a potential biomarker for periodontitis.
Smiga et al., 2023 [7]	Exp in vitro	N: N/A (no human participants)	P. gingivalis cultured under heme/iron limitation. Mutants (HmuY/HusA) tested for virulence.	*P. gingivalis *increases virulence under heme/iron limitation (p < 0.0001), and loss of key proteins (HmuY/HusA) significantly reduces host cell infection and invasion (p < 0.05).
Zeng et al., 2021 [8]	Cross-sectional	N (Exp / Ctrl): 90 / 30	Subgingival plaque sequencing across periodontal stages.	Relative abundance of the red complex significantly increased from stage II to stage IV periodontitis (p < 0.05).
Schacher et al., 2007 [9]	Compare clinical	N (Exp / Ctrl): 40 / 40	Subgingival plaque analyzed by PCR for A. actinomycetemcomitans	*A. actinomycetemcomitans *detected at significantly higher levels in aggressive periodontitis compared to chronic periodontitis (p = 0.01).
Yu et al., 2024 [10]	Cross-sectional	N (Exp / Ctrl): 50 / 50	Plaque samples were analyzed for microbial composition and diversity.	No microbial diversity difference, but microbial composition was significantly distinct (p < 0.05) in patients with mild vs. severe periodontitis. Positively associated with increased systemic inflammatory markers in stages III and IV
Schulz et al., 2019 [11]	Case- control	N (Exp / Ctrl): 60 / 60	Subgingival plaque sequenced. Compared healthy vs periodontitis	Disease is associated with higher abundance of Bacteroidetes, Spirochaetes, and Synergistetes, while healthy microbiomes show more Proteobacteria, Firmicutes, and Actinobacteria. Key pathogens (*P. gingivalis, T. denticola, T. forsythia*) and Filifactor alocis were strong biomarkers of disease
Yost et al., 2015 [12]	Cross-sectional	N = 10 (no control group separation)	Subgingival plaque microbiome analyzed with qPCR	Red complex bacteria (*P. gingivalis, T. forsythia, T. denticola*) show strong positive correlations with disease severity and clinical measures like attachment loss.
Wang et al., 2023 [13]	Cross-sectional	N (Exp / Ctrl): 64 / 32	Plaque microbiome profiled. Bacterial interaction networks analyzed.	Microbial composition and virulence are strongly influenced by bacterial interactions. Low *S. cristatus* : *P. gingivalis* ratios associated with more pathogenic microbial communities, increased diversity of antibiotic resistance genes, and greater virulence potential.
Wu et al., 2023 [14]	Cross-sectional	N (Exp / Ctrl): 40 / 40	Plaque and saliva were analyzed for microbiome and metabolomics.	*Porphyromonas gingivalis *was strongly associated with periodontitis, while shifts in carbohydrate metabolism and reduced activity of metabolic genes were observed in diseased individuals. Specific metabolites (N1-acetylspermine) and microbial changes may serve as biomarkers.
Choi et al., 2023 [15]	Cross-sectional	N (Exp / Ctrl): 80 / 40	Oral microbiota screened for P. gingivalis prevalence.	Studies show prevalence of *P. gingivalis *can reach ~79% in periodontitis vs. ~25% in healthy individuals.
Ram-Mohean et al., 2020 [16]	Cross-sectional	N (Exp / Ctrl): 25 / 25	Metatranscriptomic analysis of oral microbiota.	~20% of differentially expressed activity comes from known pathogenic complexes, while ~50% originates from previously unaffiliated organisms.
Arredondo et al., 2026 [17]	Cross-sectional	N (Exp / Ctrl): 100 / 50	Plaque microbiome sequenced for ARGs.	Periodontitis showed increased ARG richness, broader resistance, and enrichment of β-lactam, tetracycline, and aminoglycoside resistance genes; red-complex pathogens correlated with ARG abundance.
de Andrade et al., 2021 [18]	Cross-sectional	N (Exp / Ctrl): 40 / 40	Plaque samples were analyzed in overweight vs normal-weight participants.	Overweight individuals showed higher levels of periodontal pathogens (*P. gingivalis, T. forsythia*) and associations with *T. denticola*, suggesting obesity promotes early dysbiotic shifts before disease onset.
Bronzato et al., 2025 [19]	Cross-sectional	N = 72 (no clear Exp/Ctrl groups reported)	Periapical lesion microbiome profiled by sequencing.	Periapical lesions exhibited heterogeneous, population-specific microbiomes influenced by geographic and clinical factors, rather than a single pathogenic profile.
Britos et al., 2025 [20]	Cross-sectional	N (Exp / Ctrl): 56 / 28	Subgingival plaque and systemic samples were analyzed.	Periodontitis was associated with higher levels of key pathogens (*P. gingivalis, P. endodontalis, F. nucleatum*) and increased dysbiosis, with evidence of bacterial DNA translocation linking oral infection to systemic inflammation.
Chahboun et al., 2015 [21]	Cross-sectional	N (Exp / Ctrl): 30 / 30	Subgingival plaque was compared between aggressive and healthy.	Aggressive periodontitis was associated with higher levels of key pathogens (*A. actinomycetemcomitans, P. gingivalis, T. forsythia*) and distinct microbial profiles, supporting a polymicrobial disease pattern.
Damgaard et al., 2019 [22]	Cross-sectional	N (Exp / Ctrl): 55 / 55	Salivary microbiomes were analyzed.	Salivary *P. gingivalis *was associated with both aggressive and chronic periodontitis and may serve as a disease biomarker, but it did not distinguish between disease types.
Daneshmand et al., 2002 [23]	RCT	N (Exp / Ctrl): 20 / 20	Scaling and root planning ± chlorhexidine. Microbial load measured.	Periodontal pathogens decreased in both groups, but adjunctive chlorhexidine provided no significant additional antimicrobial benefit beyond scaling and root planing alone.
Balan et al., 2023 [24]	Cross-sectional	N (Exp / Ctrl): 50 / 50	Plaque and saliva were analyzed for microbiome and metabolic changes.	Periodontitis was associated with pathogenic microbial enrichment and metabolic changes, linking oral dysbiosis to systemic inflammation and Type 2 diabetes.
Fine et al., 2024 [25]	RCT	N (Exp / Ctrl): 60 / 60	Stannous fluoride applied. Inflammation and microbial load were measured.	Stannous fluoride reduced inflammation, immune activity, and periodontal pathogens, supporting the prevention of disease progression.
Gao et al., 2026 [26]	Prospective clinical	N = 90 (no clear Exp/Ctrl split)	Third-molar extraction. Pre- and post-extraction plaque sampling.	Third-molar extraction improved periodontal outcomes, reduced pathogens (P. gingivalis, P. nigrescens, F. nucleatum), and decreased inflammation, supporting better treatment response.

Risk of Bias Assessment

The methodological quality of the included studies was evaluated using study-design-specific assessment tools. Observational studies, including cross-sectional, case-control, and cohort designs, were assessed using the Newcastle-Ottawa Scale (NOS) [27], which evaluates study quality based on selection, comparability, and outcome/exposure domains. Randomized controlled trials were evaluated using the Revised Cochrane Risk of Bias tool version 2.0 (RoB 2.0; The Cochrane Collaboration, London, UK) [27, 28], which assesses bias across domains, including the randomization process, deviations from intended interventions, missing outcome data, measurement of outcomes, and selection of reported results. The in vitro study was not assessed using the Newcastle-Ottawa Scale or RoB 2.0, as these tools are designed for clinical and observational human studies. Instead, it was evaluated qualitatively based on experimental design, reproducibility, control conditions, and validity of outcome measurements. Results for the NOS assessments are shown in Table 3, and for the RoB assessments in Table 4.

**Table 3:** Quality Assessment of Observational Studies Using the Newcastle-Ottawa Scale Observational studies were evaluated using the Newcastle-Ottawa Scale (NOS) [27], which assesses study quality across three domains: selection (0–4 stars), comparability (0–2 stars), and outcome/exposure (0–3 stars), with a maximum score of 9. Studies were categorized as low (0–3), moderate (4–6), or high quality (7–9).

Study	Year	Study Design	Tool	Selection (0–4)	Comparability (0–2)	Outcome/Exposure (0–3)	Total (0–9)	Overall
Doğan et al. [5]	2003	Cross-sectional	NOS	3	1	2	6	Moderate
Jung et al. [6]	2024	Cross-sectional	NOS	3	1	3	7	High
Zeng et al. [8]	2021	Cross-sectional	NOS	4	1	3	8	High
Schachner et al. [9]	2007	Clinical comparative	NOS	3	1	2	6	Moderate
Yu et al. [10]	2024	Cross-sectional	NOS	3	1	3	7	High
Schulz et al. [11]	2019	Case-control	NOS	4	2	3	9	High
Yost et al. [12]	2015	Cross-sectional	NOS	1	0	2	3	Low
Wang et al. [13]	2023	Cross-sectional	NOS	3	1	3	7	High
Wu et al. [14]	2023	Cross-sectional	NOS	3	1	3	7	High
Choi et al. [15]	2023	Cross-sectional	NOS	2	0	2	4	Moderate
Ram-Mohan et al. [16]	2020	Cross-sectional	NOS	2	0	3	5	Moderate
Arredondo et al. [17]	2026	Cross-sectional	NOS	4	1	3	8	High
Andrade et al. [18]	2021	Cross-sectional	NOS	3	1	2	6	Moderate
Bronzato et al. [19]	2025	Cross-sectional	NOS	2	0	2	4	Moderate
Britos et al. [20]	2025	Cross-sectional	NOS	3	1	3	7	High
Chahboun et al. [21]	2015	Cross-sectional	NOS	3	1	2	6	Moderate
Damgaard et al. [22]	2019	Cross-sectional	NOS	3	1	2	6	Moderate
Balan et al. [24]	2023	Cross-sectional	NOS	3	1	3	7	High
Gao et al. [26]	2026	Prospective clinical	NOS	4	1	3	8	High

**Table 4:** Risk of Bias Assessment of Randomized Controlled Trials and In Vitro Study Randomized controlled trials (RCTs) were evaluated using the Revised Cochrane Risk of Bias Tool (RoB 2.0) [28], which assesses bias across domains including randomization, deviations from intended interventions, missing outcome data, outcome measurement, and selection of reported results. The in vitro study was not assessed using RoB 2.0, as this tool is not applicable to laboratory-based studies, and was instead evaluated qualitatively based on methodological rigor, control conditions, and validity of outcome measurements.

Study	Year	Study Design	Tool	Randomization	Deviations	Missing Data	Measurement	Reporting	Overall
Daneshmand et al. [23]	2002	RCT	RoB 2	Low	Some concerns	Low	Low	Some concerns	Moderate
Fine et al. [25]	2023	RCT	RoB 2	Low	Low	Low	Low	Low	Low
Smiga et al. [7]	2023	In vitro	Qualitative assessment	N/A	N/A	Low	High	N/A	High

Discussion

Microbial Dysbiosis and Its Role in Periodontal Disease Initiation

Periodontal disease, a chronic inflammatory condition affecting the structures and supporting tissues of the teeth, is strongly associated with changes in the oral microbiome. From symbiosis to dysbiosis, changes in microbial composition, rather than overall diversity, are strongly influential in this chronic condition. Research across several studies has reported no significant difference in alpha diversity between healthy and diseased individuals. However, in studies reporting beta diversity, differences in beta diversity indicate that microbial composition, the type of bacteria present, plays a more important role in periodontal disease progression, rather than the bacterial load, the amount of bacteria present [10, 11]. These findings support the concept that periodontal disease is a polymicrobial condition that is strongly driven by ecological imbalance rather than the presence of a single pathogen.

Key Periodontal Pathogens and Their Association with Disease Severity

A consistent pattern across the literature is the increasing abundance of key pathogenic species with disease severity, particularly members of the red complex, including *Porphyromonas gingivalis*, *Tannerella forsythia*, and *Treponema denticola*. These organisms were found to be significantly more prevalent in periodontitis than in healthy controls and showed strong correlations with clinical parameters, including probing depth and clinical attachment loss [12, 13]. For example, *P. gingivalis *and *T. forsythia *showed a significant positive correlation with attachment loss (r = 0.62, p < 0.001), indicating their direct involvement in disease progression [12]. Additionally, detection rates of these pathogens increased stepwise with disease severity, with *P. gingivalis *rising from 16% in mild cases to 76% in severe cases, and *T. forsythia *increasing from 24% to 79%, reinforcing their role as indicators of disease advancement [13].

Community-Level Microbial Interactions in Disease Progression

The progression of periodontal disease is also greatly attributed to broader shifts in microbial communities and the bacteria’s roles. Transcriptomic analyses revealed that only a minority of microbial activity originates from traditionally recognized pathogenic complexes, with approximately 50% of activity derived from previously unaffiliated organisms, highlighting the importance of community-wide interactions in disease progression [16]. Furthermore, microbial co-occurrence networks become increasingly disrupted with increasing disease severity, resulting in reduced stability and greater vulnerability in the advanced stages of periodontitis. These findings suggest that periodontitis progression is driven not only by specific pathogens but also by the breakdown of key microbial interactions that maintain oral health.

Periodontal Pocket Environment and Pathogen Adaptation

Changes in the environment within the periodontal pocket further contribute to disease progression. Deepening periodontal pockets create anaerobic environments that promote the growth of infectious pathogens like *P. gingivalis *and *T. forsythia*. These species not only increase in abundance but can also become more aggressive under certain conditions, such as limited heme or iron availability, thereby helping hosts invade surrounding tissues and drive disease progression [7]. Additionally, the relative abundance of red complex bacteria has been shown to increase significantly from early to advanced disease stages, while health-associated species like *Streptococcus *decrease. This is reflected in a transition toward a pathogenic microbiome [8].

Microbial Variability Across Periodontal Disease Phenotypes

Despite the link between red complex bacteria and disease progression, variability exists by disease type. For instance, *Aggregatibacter actinomycetemcomitans *was significantly more prevalent in aggressive periodontitis than in chronic periodontitis (p = 0.01), suggesting that distinct microbial profiles may contribute to different disease phenotypes [9]. Similarly, some studies have reported that microbial diversity remains unchanged across disease stages, emphasizing that shifts in bacterial species are a stronger indicator of progression than overall diversity [10].

Multifactorial Nature of Periodontal Disease and Clinical Implications

Ultimately, periodontal disease is a multifactorial process influenced by microbial dysbiosis, increased abundance of key pathogenic species, and disruption of microbial community structure. The association between specific bacterial profiles and disease severity highlights that the oral microbiome could serve as an important tool for future diagnostics and treatment.

Microbial Composition as a Determinant of Periodontal Disease

Across the studies reviewed, periodontal disease consistently emerged as a condition driven by alterations in microbial community composition rather than infection by a single etiologic pathogen. Both classical culture‑based investigations and modern high‑resolution sequencing analyses demonstrated that disease is associated with qualitative changes in microbial balance rather than absolute microbial load. There was early evidence of this paradigm, showing that periodontal pockets measuring 6-7 mm harbored complex microbial communities dominated by Gram-negative anaerobes, including *Porphyromonas gingivalis *and *Tannerella forsythia *[23]. Although scaling and root planning resulted in a significant reduction in total bacterial colony counts over four weeks (p = 0.005), the relative proportions of major periodontal pathogens were not significantly different between sites treated with scaling alone versus those receiving adjunctive chlorhexidine chips (p ≈ 0.92) [23]. These findings emphasize the resilience of the dysbiotic biofilm and suggest that periodontal disease control depends on ecological disruption rather than selective antimicrobial suppression.

Red Complex Pathogens and Disease Severity

A consistent observation across the literature was the enrichment of red‑complex organisms with increasing disease severity. Salivary *P. gingivalis *was detected in 64.5% of patients with aggressive periodontitis and 52% of patients with chronic periodontitis, compared with only 8% of periodontally healthy controls (p < 0.001) [22]. Receiver‑operating characteristic analyses demonstrated strong discriminatory capacity for detecting disease presence, with areas under the curves (AUCs) of 0.80 for aggressive periodontitis and 0.72 for chronic periodontitis. Importantly, no statistically significant difference in *P. gingivalis *prevalence or relative abundance was observed between aggressive and chronic forms of periodontitis (p = 0.42), reinforcing the concept that red‑complex organisms serve as indicators of advanced dysbiosis rather than determinants of disease phenotype [22]. Comparable trends were observed in subgingival microbiome analyses reported in [26], in which red‑complex species persisted or re‑emerged at sites that maintained anaerobic ecological conditions despite therapy.

Aggregatibacter Actinomycetemcomitans *and Aggressive Disease Phenotypes*

In contrast to red‑complex pathogens, *Aggregatibacter actinomycetemcomitans *demonstrated a more specific association with aggressive disease phenotypes. A report that *A. actinomycetemcomitans *was detected in 60% of aggressive periodontitis patients compared with only 25% of chronic periodontitis patients, with mean proportions significantly higher in generalized aggressive disease (11.55% vs. 0.05%; p = 0.004) [21]. These findings suggest that *A. actinomycetemcomitans *may play a disproportionate role during early or rapidly progressing disease. Further research has demonstrated that *Aggregatibacter *species were enriched alongside functional pathways related to iron acquisition and lipopolysaccharide synthesis, indicating that virulence potential may be mediated through metabolic activity rather than abundance alone [24]. Collectively, these data highlight variability in microbial profiles at disease onset and underscore the role of host susceptibility and ecological context.

Microbial Diversity and Community Structure

Despite pronounced changes in the abundance of pathogenic species, microbial diversity metrics remained relatively stable between health and disease. Reports of comparable Shannon diversity indices among aggressive periodontitis (3.4), chronic periodontitis (3.4), and healthy controls (3.5) indicate that periodontal disease is not characterized by reduced microbial richness [22]. Instead, beta‑diversity analyses revealed distinct clustering of microbial communities by disease status, reflecting structural reorganization rather than diversity loss. This observation was corroborated in a longitudinal setting, demonstrating significant divergence in subgingival microbial community structure between sites treated with or without third‑molar extraction at both three and six months post‑therapy (p = 0.001), despite only modest decreases in alpha diversity [26]. These findings strengthen the interpretation that periodontal disease reflects a dysbiotic shift in microbial balance rather than microbial depletion.

Functional Dysbiosis and Metabolic Reprogramming

Functional profiling further elucidates the pathogenic nature of dysbiotic microbial communities. Using whole‑metagenome shotgun sequencing, identified significant enrichment of metabolic pathways involved in lipopolysaccharide biosynthesis, ferroptosis, iron homeostasis, amino‑acid metabolism, and fatty‑acid biosynthesis among periodontitis patients [24]. Several lipopolysaccharide‑related pathways remained statistically significant after false‑discovery‑rate correction, indicating heightened inflammatory potential. Notably, many taxa contributing to these pathways formed part of a shared core microbiome present in both health and disease, underscoring that pathogenicity arises from altered functional expression rather than exclusive colonization by specific organisms. This metabolic reprogramming provides a mechanistic explanation for sustained inflammation and tissue destruction observed clinically.

Environmental Modification and Microbial Shifts

Clinical interventions that alter the local periodontal environment further support an ecological model of disease. Demonstrations showed that removal of adjacent non‑impacted third molars resulted in significantly greater probing depth (PD) reductions when combined with non‑surgical periodontal therapy compared to therapy alone (change in PD (ΔPD)** **= 1.06 ± 0.76 mm vs. 0.52 ± 0.69 mm at three months; p < 0.001) [26]. These clinical improvements were accompanied by significant reductions in *Porphyromonas gingivalis*, *Prevotella nigrescens*, and *Fusobacterium nucleatum*, alongside enrichment of health‑associated taxa such as *Neisseria oralis*. These findings indicate that modification of anaerobic niches and plaque retention sites can shift microbial communities toward a less pathogenic configuration, reinforcing the importance of local ecological conditions in disease persistence.

Host-Microbe Modulation and Therapeutic Implications

Host‑directed interventions also demonstrate substantial effects on microbial ecology and inflammatory regulation. Showing that use of a stabilized stannous fluoride dentifrice during experimental gingivitis significantly reduced bleeding on probing, gingival index scores, and oral neutrophil counts compared to sodium fluoride controls (p < 0.05) [25]. Gingival crevicular fluid levels of matrix metalloproteinase-8 (MMP-8)​​​​​​​ and receptor activator of nuclear factor kappa-B ligand (RANKL)were also significantly lower in the treatment group, reflecting suppression of tissue‑destructive inflammatory pathways. Microbiome analyses revealed significant reductions in gram‑negative genera, including Porphyromonas, Tannerella, and Treponema, without complete elimination of these organisms. These findings further support the notion that effective periodontal management involves modulation of host‑microbe interactions rather than eradication of individual pathogens.

Integrated Interpretation

The studies reviewed demonstrate that periodontal disease progression reflects a coordinated transition from microbial symbiosis to dysbiosis. Disease severity correlates with structured shifts in microbial composition, enhanced inflammatory metabolic activity, and altered environmental conditions rather than the presence of a single causative organism. Red‑complex pathogens increase with disease progression; *Aggregatibacter actinomycetemcomitans *shows stronger associations with aggressive phenotypes; and overall microbial diversity remains relatively stable, while community structure and function undergo significant change. These converging findings emphasize the importance of understanding periodontal disease as an ecological imbalance, with implications for improving diagnostics and developing targeted therapeutic strategies centered on restoring microbial homeostasis.

Limitations and future directions

This research is limited by diversity across the included studies in disease classification systems, sequencing platforms, and sampling methods, making direct comparisons difficult. Many studies used different periodontal staging and grading criteria and requirements, which can lead to inconsistencies in how disease severity and progression were defined. Additionally, many of the included microbiome studies had a variety of sample types, such as saliva, subgingival plaque, or gingival crevicular fluid, which could influence bacterial detection and relative abundance. There is also a predominance of cross-sectional designs, which do not allow conclusions about temporal changes or causal pathways in disease onset. Several laboratory studies focused on mechanistic pathways may not translate to clinical conditions, further limiting generalizability. Finally, publication bias remains a strong possibility, as studies showing significant microbial associations are more likely to be published than studies with null results.

## Conclusions

The findings from the research strongly support the conclusion that periodontal disease is not driven by the presence of a single pathogen but by microbial dysbiosis. Consistent trends across the literature demonstrate that the incidence and abundance of key periodontal pathogens, particularly members of the red complex (*P. gingivalis*, *T. forsythia*, and *T. denticola*), increase with disease severity and closely correlate with clinical indicators of progression. Additionally, Aggregatibacter actinomycetemcomitans shows a stronger association with aggressive disease phenotypes, demonstrating the variability in microbial profiles at disease onset. Importantly, microbial diversity remains relatively stable, whereas shifts in microbial composition are a more reliable indicator of disease progression. As periodontal disease advances, disruptions in microbial community structure and function, along with environmental changes within the periodontal pocket, further promote pathogenic activity. These findings align with the earlier concept that disease results from a transition from symbiosis to dysbiosis in the oral microbiome. Ultimately, understanding these microbial shifts is essential for improving diagnostic approaches and developing more targeted therapeutic strategies.
